# A Rare Case of Extracavitary Primary Effusion Lymphoma in the Bladder and Ureter

**DOI:** 10.1155/2020/6124325

**Published:** 2020-01-19

**Authors:** Jiankun Tong, Sana Jadallah, William H. Rodgers, Gabriel Jung, Malvina Fulman, Abhisek Swaika

**Affiliations:** ^1^Department of Pathology, New York–Presbyterian Queens, 56-45 Main Street, Flushing, NY 11355, USA; ^2^Weill Cornell Medical College, 525 East 68th Street, Box 130, New York, NY 10065, USA; ^3^Hematology & Medical Oncology, Queens Medical Associates, 176-60 Union Tpke # 360, Fresh Meadows, NY 11366, USA

## Abstract

Primary effusion lymphoma (PEL) is a rare and very aggressive large B-cell lymphoma usually presenting as serous effusions without a tumor mass. It is universally associated with human herpesvirus type-8 (HHV-8) infection. It most commonly occurs in the body cavities and rarely develops as solid tumor masses in the wall of cavity and other organs, and it has been termed as extracavitary PEL. Extracavitary PEL has been reported in the lymph nodes and extranodal sites. Here we report a rare case of extracavitary PEL occurring in the bladder and ureter of a human immunodeficiency virus (HIV)-negative 76-year-old Chinese male, presenting with right leg swelling, erythema, and pain. To the best of our knowledge, this is the first case of extracavitary PEL presenting in the bladder and ureter.

## 1. Case Presentation

A 76-year-old Chinese male, former smoker, with no other significant past medical history or other high-risk behavior and not on medications presented to the hospital for worsening right lower extremity swelling, erythema, and pain. The swelling suddenly started 3 days ago, with occasional cough and dyspnea on exertion. He denied trauma or injury to the area, fever, chills, chest pain, nausea, vomiting, abdominal pain, and dysuria. He did mention some unintentional weight loss. Physical exam was pertinent for right leg swelling, decreased breath sounds on right side, and abdominal distension.

Laboratory analysis revealed mild leukocytosis with normal differentials, mild anemia (Hb 10.6 g/dl), and a normal platelet count of 386 K/*μ*l. His creatinine was elevated. HIV and hepatitis testing were negative. Epstein–Barr virus (EBV) serology signified a past infection. HHV-8 serology testing was not available. Bilateral Doppler ultrasound results were negative for deep vein thrombosis, and a ventilation-perfusion (VQ) scan revealed a low probability for pulmonary embolism.

**Figure 1:**
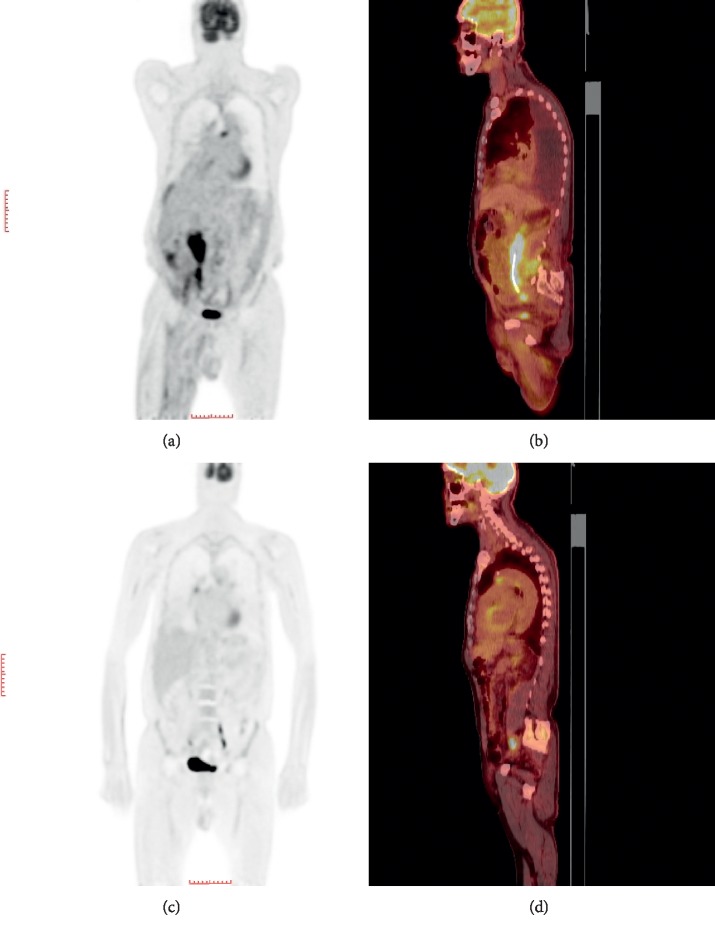
Initial diagnostic PET-CT scan with soft tissue mass encasing the right proximal ureter (a and b). Complete resolution of right periureteral mass on treatment completion PET-CT scan (c and d).

Renal ultrasound demonstrated moderate hydronephrosis on the right and mild hydronephrosis on the left, with no evidence of calculi in the kidney, ureters, and bladder. Computed tomography (CT) and a following positron emission tomography (PET) scan revealed a mass like encasement of the proximal right ureter and bladder (Figures 1(a) and 1(b)), along with retroperitoneal lymphadenopathy on the right side. Moderate to large pleural effusion was noted on the chest CT scan. Subcutaneous lymphedema was seen on magnetic resonance imaging (MRI) of the right lower extremity. His ejection fraction was normal on echocardiogram. Patient refused a lumbar puncture. He did not have any neurological symptoms.

Urine cytology revealed atypical urothelial cells. Patient underwent cystoscopy and right ureteroscopy, demonstrating bullous edema and erosion at the bilateral trigone areas and in the right mid-distal ureter. Bladder biopsy was taken and a right ureteral stent was placed. The patient also underwent a diagnostic and therapeutic thoracentesis.

## 2. Diagnosis

**Figure 2:**
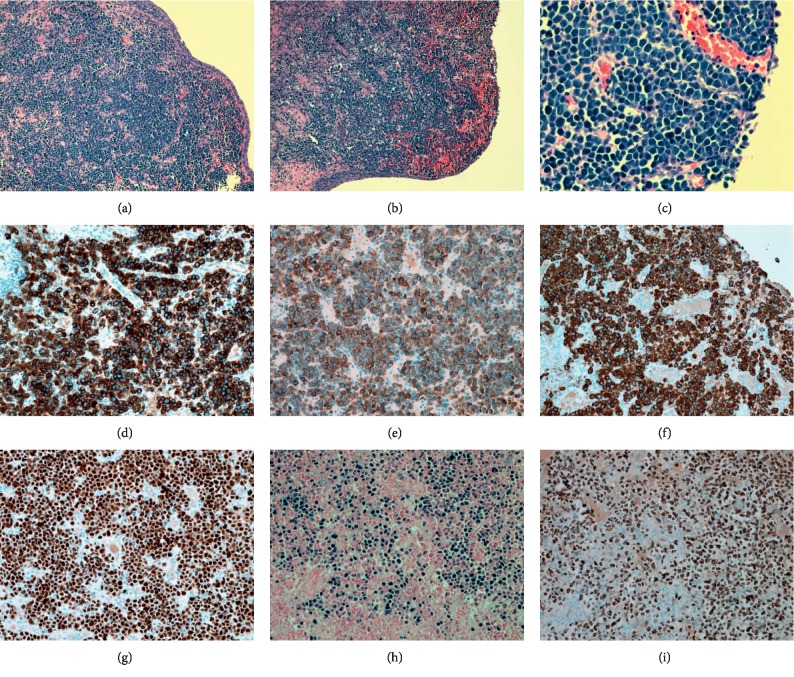
HE section shows the neoplastic cells in the suburothelium (×2) (a). Urothelial mucosa in bladder shows focal mucosal erosion and ulceration (×2) (b). HE section shows medium-sized to large neoplastic cells (×40) (c). The neoplastic cells are positive for CD30 (d), CD138 (e), EMA (f), MUM-1 (g), EBER (h), and HHV-8 (i).

Microscopic examination of the histologic sections showed sheets of medium-sized to large neoplastic cells in the suburothelium (Figures 2(a) and 2(b)) with scant cytoplasm, a high nuclear/cytoplasmic (N/C) ratio, round or oval nuclei with fine or vesicular chromatin, and indistinct nucleoli (Figure 2(c)). Mitotic figures and apoptotic bodies were frequently seen. The urothelial mucosa demonstrated focal erosion and hemorrhage (Figure 2(b)).

An extensive immunohistochemistry (IHC) panel was performed which found the neoplastic cells to be positive for CD30 (Figure 2(d)), CD45 (weak), CD138 (Figure 2(e)), EMA (Figure 2(f)), MUM-1 (Figure 2(g)), EBER (Figure 2(h)), and human herpesvirus 8 (HHV-8) (Figure 2(i)), while being negative for AE1/3, ALK-1, BCL-1, BCL-6, CAM 5.2, CD3, CD5, CD10, CD20, CD34, CD68, CD79a, CD99, chromogranin, desmin, HMB-45, kappa and lambda, Mart-1, PAX-5, S100, synaptophysin, TdT, and WT-1.

**Figure 3:**
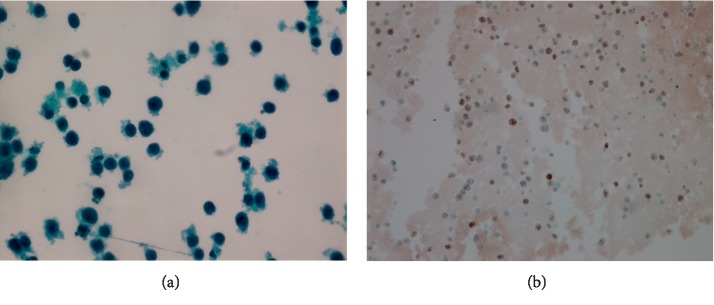
The thin-prep shows large atypical hematopoietic cells in the pleural fluid (×40) (a). The neoplastic cells are positive for HHV-8 (b).

The thin-prep and cell block analysis on the pleural fluid also demonstrated the large atypical hematopoietic cells (Figure 3(a)) and had a similar immunophenotype profile to that of the bladder biopsy, also being positive for HHV-8 (Figure 3(b)).

The consistent morphology and immunophenotypic characteristics along with the clinical presentation led to the diagnosis of an extracavitary PEL of the bladder and ureter.

## 3. Treatment

Due to the aggressive nature of this subtype of lymphoma, International Prognostic Index (IPI) score 5, the patient was promptly started on chemotherapy with cyclophosphamide, Adriamycin, vincristine, and prednisone (CHOP), outside of a clinical trial. He tolerated the treatment well and had a continued significant clinical and radiological response to chemotherapy. His breathing improved, and he did not require any further thoracentesis. He gained weight, and his creatinine also normalized, undergoing an interim stent exchange. An interim PET scan after 2 cycles demonstrated decreasing bladder wall thickness and size of the periureteral mass and lymphadenopathy, along with reduced pleural effusion. He continued on the CHOP chemotherapy and completed 6 cycles attaining almost complete radiological remission on the following PET-CT scan (Figures 1(c) and 1(d)).

He was started on active surveillance. Due to the initial advanced stage presentation (lymphomatous pleural effusion) and complete radiological response to systemic chemotherapy, radiation therapy was not considered especially due to the site of disease presentation and the paucity of clinical data. Unfortunately, he relapsed within 3 months with a recurrent periureteral mass, abdominal lymphadenopathy, and neck lesions. Repeat biopsy of the neck lesion confirmed the extracavitary PEL diagnosis with the same IHC profile as prior (HHV-8+, CD 30+, EBER+, MUM-1+, and CD 20−). Kaposi sarcoma and multicentric Castleman disease were again ruled out given the HHV-8 positivity. He was deemed to not be a transplant candidate. With the paucity of available and accessible appropriate clinical trial for an HIV-negative rare PEL presentation, the patient was started on salvage brentuximab vedotin (CD-30+) and then bendamustine chemotherapy, outside of a clinical trial. The patient again had very abbreviated clinical and radiological responses to the treatments, but thereafter could not tolerate any further antineoplastic treatments. He was placed in hospice, and he passed away due to this aggressive lymphoma.

## 4. Discussion

Primary effusion lymphoma is a rare and very aggressive large B-cell lymphoma usually presenting as malignant effusions without a tumor mass. It usually occurs in the body cavities, and the most common sites are the pleural, pericardial, and peritoneal cavities [1–3]. Most cases are limited to the body cavities of origin, but subsequent dissemination can happen. It rarely develops as solid tumor masses in the wall of cavity and other organs and has been termed as extracavitary PEL. Extracavitary PEL has been reported in the lymph nodes, the most frequent site, and few extranodal sites, such as the central nervous system, intestinal tract, lungs, and skin [1–7], similar to the other extranodal noncavitary non-Hodgkin lymphoma (NHL) presentations.

PEL accounts for approximately 4% of HIV-associated non-Hodgkin lymphoma and <1% of non-HIV-related lymphomas [8]. There is a frequent coinfection of EBV, accounting for 60%–90% of cases [9–11]. PEL and extracavitary PEL are universally associated with human herpesvirus type-8 (HHV-8) infection [9, 12].

Diagnosis of PEL and extracavitary PEL is based on the clinical manifestation along with morphology, immunohistochemistry, and molecular studies.

On histopathological analysis, the lymphoma cells exhibit a variety of appearances, ranging from large immunoblastic/plasmablastic cells to cells with anaplastic morphology [13]. The IHC is typically positive for CD30, CD38, CD45, VS38c, CD138, HHV-8, and MUM-1. EMA is variably expressed on the lymphoma cells, but they usually lack the expression of B-cell markers (CD19, CD20, CD79a, surface and cytoplasmic immunoglobulin) and T-cell markers (CD2, CD3, CD4, CD5, CD7, and CD8) [13–17]. The definitive/confirmatory diagnosis of PEL requires the detection of HHV-8 infection in lymphoma cells, which can be performed by immunohistochemistry, PCR, or molecular studies [14].

PEL is clinically unique as it predominantly presents as a lymphomatous malignant effusion within body cavities, hence confirming the diagnosis, whereas the diagnosis of extracavitary PEL is challenging since it behaves variably at the different primary sites or precedes the classic variant of PEL. By no difference, the histopathological feature of extracavitary PEL is similar to PEL thereby aiding with the diagnosis [13], though other differentials need to be diligently excluded.

The most common differential diagnosis includes other subtypes of aggressive non-Hodgkin lymphoma such as plasmablastic lymphoma, anaplastic large-cell lymphoma, diffuse large B-cell lymphoma, and plasmacytoma with anaplastic variant. Other malignant tumors such plasmacytoid urothelial carcinoma, and malignant melanoma also warranted exclusion in this case. Although each of these differential diagnoses mentioned above shares morphologic features and some immunophenotypic profile with PEL and extracavitary PEL, each can be easily distinguished from PEL by the absence of HHV-8 antigen within neoplastic cells.

PEL is most commonly seen in immunocompromised patients, such as patients with concomitant HIV infection, organ transplant, etc. As in other lymphomas, active treatment of the HIV infection, reversal of the immune suppression, takes precedence along with antilymphoma treatment as demonstrated in a recent case study of prolonged benefit of liposomal doxorubicin in an HIV infected PEL patient [18]. PEL can, however, rarely present in patients with an intact immune function, like in our patient. Due to the rarity of this malignancy, treatment recommendations are generally based on expert opinions, retrospective case series, reports, etc., and hence no consensus treatment guideline exists. Enrollment in well designed and prospective clinical trial is understandably challenging.

Initial treatment consists of use of standard aggressive lymphoma chemotherapy regimens such as dose adjusted EPOCH or CHOP. The lack of CD-20 expression precludes any benefit of rituximab use. Patients with relapsed disease are treated similar to other aggressive lymphomas and should be evaluated for autologous stem cell transplant. More emphasis should be placed on an expedited diagnosis and early enrollment in clinical trials.

To the best of our knowledge, this is the first case of an extracavitary PEL which occurred in the bladder and ureter, along with the more classical serosal involvement of the right pleural cavity.

In summary, extracavitary PEL can clinically present as an aggressive lymphoma at any unexpected location, even in the absence of HIV infection or other forms of immune suppression. A diligent diagnostic suspicion should promptly lead to a HHV-8 antigen testing to avoid a diagnostic pitfall, and help initiate an expedited antilymphoma treatment. PEL is associated with a very poor prognosis, and clinical trial enrollment should be strongly encouraged.
